# Beyond Type B: A Rare and Challenging Case of Neonatal Meningitis

**DOI:** 10.7759/cureus.88299

**Published:** 2025-07-19

**Authors:** Abdessamad Lalaoui, Khalid Abi El Aala, Ghizlane Kassal, Fatiha Bennaoui, Nadia El Idrissi Slitine, Fadl Mrabih Rabou Maoulainine

**Affiliations:** 1 Mother and Child Department, Neonatology/General Pediatrics, Mohammed VI University Hospital Center, Marrakesh, MAR; 2 Research Center for Childhood, Health and Sustainable Development, Cadi Ayyad University, Marrakesh, MAR

**Keywords:** cerebrospinal fluid culture, meningitis, newborn, non-type b haemophilus influenza, transfontanelar ultrasound

## Abstract

*Haemophilus influenzae* (Hi) meningitis is a rare but serious bacterial infection in newborns and infants. While type b (Hib) has historically been the most prevalent strain, non-b serotypes remain uncommon and are not well characterized, underscoring the need for accurate identification and serotyping to improve clinical understanding and management. These non-b variants have been increasingly associated with severe neurological complications and poor outcomes, highlighting the importance of early recognition and targeted therapeutic intervention. We report the case of a 23-day-old newborn diagnosed with Hi meningitis caused by a non-b serotype, illustrating the complexities of this rare presentation. This case emphasizes the clinical relevance of non-type b Hi meningitis and explores the diagnostic challenges, treatment considerations, and broader implications for neonatal infectious disease research.

## Introduction

*Haemophilus influenzae* is a bacterium capable of causing a range of infections, particularly in infants and young children. Over the past four decades, the incidence of meningitis caused by *H. influenzae *in children has significantly declined, largely due to the widespread implementation of vaccination against *H. influenzae *type b (Hib). This vaccine has proven highly effective in reducing case numbers and preventing the severe complications associated with Hib infection [1,2].

However, there has been a reported increase in invasive diseases caused by *H. influenzae *strains not covered by the current vaccines [2]. Non-typeable *Haemophilus influenzae *(NTHi), a commensal organism in the upper respiratory tract of children, can cause respiratory infections such as otitis media, sinusitis, conjunctivitis, and bronchitis [3,4]. Rarely, it may lead to more severe infections such as meningitis or septicemia, particularly in children with predisposing factors such as head trauma [5], CSF leakage [6], or immunocompromised states [7]. In neonates, transmission may occur via maternal genital tract colonization [8,9], although there have been occasional reports of infection in previously healthy children [10-12].

At present, there are limited diagnostic tools available to specifically detect non-type b *H. influenzae*, posing significant challenges in the timely identification and management of infections caused by these strains [13].

We present a case that highlights the clinical and paraclinical features, disease progression, and outcome of *H. influenzae *non-type b meningitis in a newborn, a pathogen that remains relatively uncommon in our clinical experience.

## Case presentation

We report the case of a 23-day-old male newborn, delivered after a well-monitored, full-term singleton pregnancy, with no significant history of maternal or perinatal infections. The newborn demonstrated good extra-uterine adaptation; the mother confirmed an immediate postnatal cry, the birth weight was 3200 g, and no notable pathological antecedents were identified.

The infant presented with a three-day history of acute fever peaking at 39°C, accompanied by feeding refusal and episodes of milk vomiting. The clinical picture became more complex with the onset of convulsive seizures, characterized by boxing and pedaling movements, along with chewing-like motions.

On examination, the infant was gesticulating, with stable hemodynamic and respiratory parameters. Notable findings included axial and peripheral hypotonia, a weakened sucking reflex, and a bulging fontanel.

Laboratory investigations revealed signs of an inflammatory response, including a markedly elevated CRP level of 367.89 mg/L, hyperleukocytosis of 30,220 cells/µL - predominantly neutrophils (21,820 cells/µL) - and a normal platelet count (161,000 cells/µL). Blood cultures were negative (Table 1).

**Table 1:** Summary of blood count results ^*^ Normal reference values sourced from [14].

Variables	Admission	Normal range^*^
Leukocytes (cells/μL)	30.22	8.900-16.690
Neutrophils (cells/μL)	21.82	3.490-9.420
Hemoglobin (g/dL)	11.6	11.6-14.2
Platelets (cells/μL)	161	157.000-406.000
CRP (mg/dL)	367.89	<0.04

Lumbar puncture findings confirmed meningitis, with a cellularity of 620 cells/mm³ (40% neutrophils), elevated proteinorrachia at 2.25 g/L, hypoglycorrhachia at 0.25 g/L, and a low glycorrhachia-to-glycemia ratio of 0.23 (Table 2).

**Table 2:** Summary of CSF findings ^*^ Normal reference values sourced from [14].

CSF	Admission	Normal range^*^
Cellularity (cells/mm³)	620	<12
Neutrophils (%)	40	<10
Proteinorrachia (g/L)	2.25	0.68
Glycorrachia (g/L)	0.25	0.38
Glycorrachia-to-glycemia ratio	0.23	≥0.6
Culture	*Haemophilus influenzae* non-type b	-

**Figure 1:**
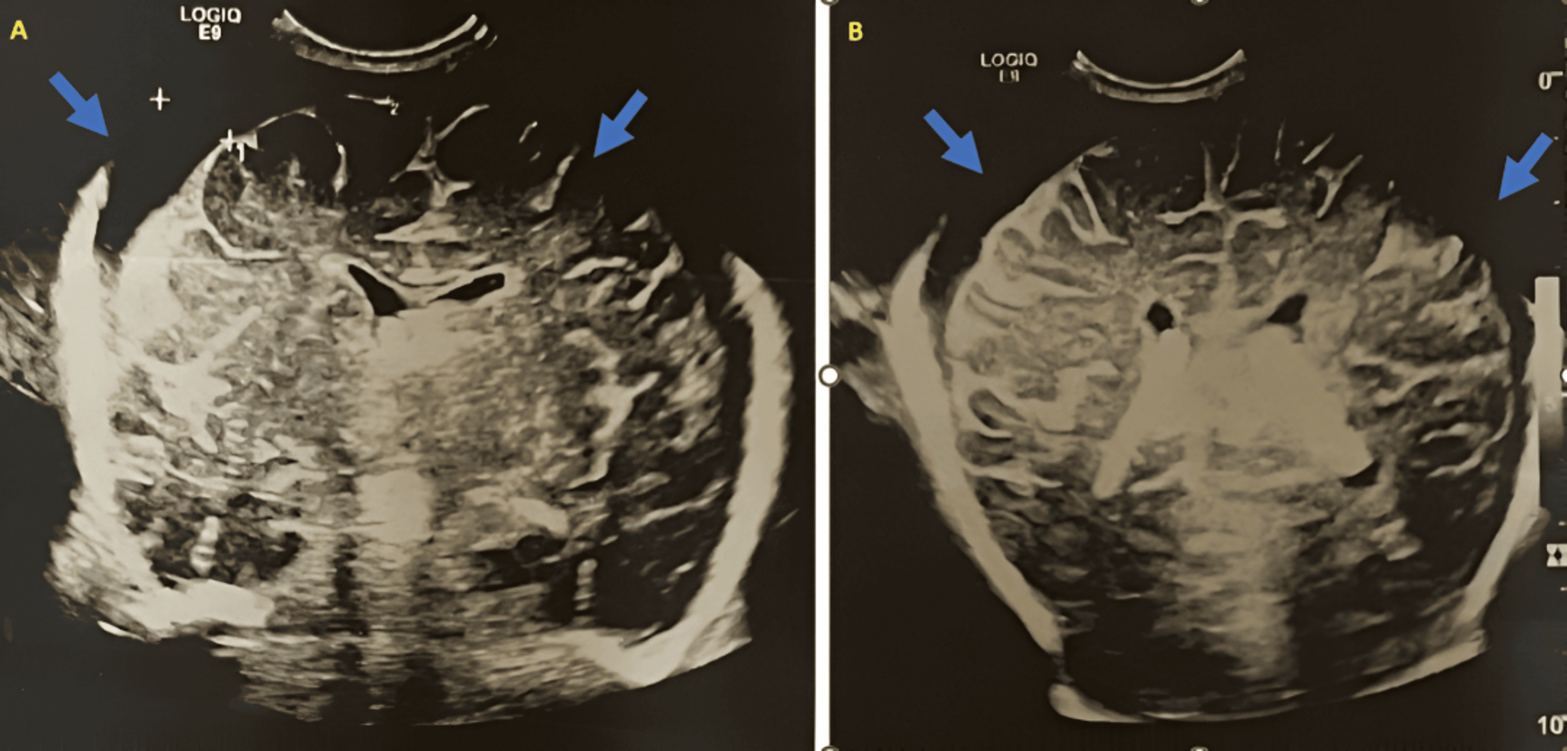
Head ultrasound showing subdural effusion (A, B) Coronal gray-scale head ultrasound images showing anechoic subdural fluid collections along the bilateral frontoparietal convexities, measuring 10 × 6 mm (blue arrows).

Empirical treatment was initiated with an intravenous meningeal dose of cefotaxime at 200 mg/kg/day, in combination with gentamycin at 5 mg/kg/day. A transfontanellar ultrasound revealed a subdural effusion along the bilateral frontoparietal convexities, as shown in Figure 1.

Within 48 hours, CSF culture on chocolate agar, supplemented by latex agglutination testing, confirmed the presence of *H. influenzae *non-type b, which was sensitive to cefotaxime. The antimicrobial regimen was continued for 21 days, and adjunctive corticosteroid therapy with dexamethasone was administered for five days. The patient showed favorable progress, with clinical examination revealing a responsive and apyretic newborn, spontaneous gesticulation, a restored sucking reflex, a normotensive fontanel, and a stable cranial perimeter.

On the 10th day of hospitalization, a follow-up transfontanellar ultrasound showed a significant reduction in the subdural collection, reflecting continued improvement. On day 41, hearing screening revealed no abnormalities. At the five-month follow-up, a transfontanellar ultrasound confirmed complete resolution of the subdural effusion.

## Discussion

*H. influenzae *primarily affects infants and young children, causing serious illnesses such as meningitis, epiglottitis, bacteremia, and pneumonia. Continuous monitoring of invasive disease is essential for evaluating the effectiveness of Hib vaccination programs and identifying emerging infections caused by non-Hib strains. Accurate cultivation of *H. influenzae *and precise serotyping of its capsular polysaccharides require advanced laboratory techniques; however, serotyping results can sometimes be inconsistent [15-19].

Since the global rollout of Hib vaccines, there has been an observed increase in the incidence of invasive non-Hib disease among children under five years old [17]. Research indicates that NTHi strains are now the most prevalent and clinically significant non-Hib types. Moreover, encapsulated non-Hib strains - serotypes a, c, d, e, and f - have begun to emerge following the introduction of Hib vaccination [16,20].

In England and Wales, the incidence of neonatal invasive NTHi disease is estimated at 2.1-4.1 per 100,000 live births, with most cases occurring in the perinatal period. Premature infants, especially those born before 28 weeks of gestation, are at markedly higher risk. Notably, most mothers of affected neonates were healthy at delivery, though they tended to be younger and more often first-time mothers compared to national averages [21]. In our case, the newborn was delivered full-term to a healthy 22-year-old mother, consistent with the demographic trends reported in the literature.

Two studies - from Italy [22] and from England and Wales [21] - have highlighted the increasing burden of invasive NTHi disease during the perinatal period. However, the current literature largely comprises case reports and small case series, which emphasize the severity of these infections in both mothers and neonates.

Serotype a (*H. influenzae *type a or Hia) is considered the second most virulent encapsulated strain after type b (Hib), sharing a genetic structure that closely resembles Hib. Cases of Hia meningitis are primarily reported in otherwise healthy children between six and 24 months of age [23-25].

In our case, the decision to initiate corticosteroid therapy was driven by the high risk of neurological complications associated with *H. influenzae *meningitis, particularly sensorineural hearing loss. Although evidence in neonates is limited, the multidisciplinary team supported corticosteroid use based on established pediatric guidelines and literature showing benefit in older infants. Mclntyre et al. [26] have reported that corticosteroids may reduce hearing loss in *H. influenzae *meningitis, while a 2015 Cochrane Review [27] acknowledges potential benefit in selected neonatal cases. In this context, corticosteroids were initiated as an adjunctive therapy to help reduce inflammation and lower the risk of long-term sequelae.

An active hospital-based surveillance study conducted in Salvador, Brazil, from 1996 to 2007, found that meningitis caused by *H. influenzae *serotypes b (Hib) and a (Hia) was associated with higher mortality rates than meningitis caused by serotypes e (Hie), f (Hif), or non-encapsulated strains.

Similarly, a US-based study underscored the severity of invasive disease caused by non-type b *H. influenzae*, which was frequently accompanied by severe complications, neurological sequelae, and increased hospitalization rates. Among children with meningitis, abnormal radiologic findings were common, and many developed serious neurological complications such as status epilepticus, subdural effusion, ventriculitis, hydrocephalus, cerebral infarction, and subdural empyema requiring drainage. Long-term outcomes included developmental delays and hearing loss [24].

In our reported case, the patient developed seizures, meningitis, and subdural effusion - all neurological complications that gradually resolved with appropriate management. Follow-up hearing screening was normal, and by the age of two, the child demonstrated normal psychomotor development, indicating a favorable recovery.

Despite the decline in invasive disease following the introduction of the Hib vaccine, *H. influenzae *remains a significant pediatric pathogen, with non-type b strains now implicated in severe illnesses. Ongoing surveillance and further research are critical to understanding the emergence of these strains and the mechanisms underlying their role in invasive disease.

## Conclusions

While the Hib conjugate vaccine has significantly reduced the incidence of Hib meningitis, it has also underscored the importance of examining capsular serotypes in all cases of *H. influenzae *meningitis. This is particularly critical because the vaccine does not offer protection against NTHi, which has emerged as a growing clinical concern. In-depth molecular characterization of NTHi is essential to better understand its genetic diversity, mechanisms of antibiotic resistance, and pathogenic features, especially among strains associated with neonatal infections.

To address the rising threat of NTHi and strengthen preventive strategies, the integration of advanced genomic tools and molecular profiling will be vital. Renewed efforts to identify alternative vaccine candidates and develop targeted antimicrobial therapies could substantially enhance global initiatives to combat *H. influenzae*-related infections and provide better protection for vulnerable populations.

## References

[1] Barbour ML (1996). Conjugate vaccines and the carriage of Haemophilus influenzae type b. Emerg Infect Dis.

[2] Ito T, Shibata H, Nakazawa M, Myokai M, Ikegaya K, Tsuchiya K, Kamimaki T (2011). Meningitis and septicemia caused by nontypeable Haemophilus influenzae in a previously healthy 2-year-old girl. J Infect Chemother.

[3] Murphy TF, Faden H, Bakaletz LO (2009). Nontypeable Haemophilus influenzae as a pathogen in children. Pediatr Infect Dis J.

[4] O'Neill JM, St Geme JW 3rd, Cutter D, Adderson EE, Anyanwu J, Jacobs RF, Schutze GE (2003). Invasive disease due to nontypeable Haemophilus influenzae among children in Arkansas. J Clin Microbiol.

[5] Kunze W, Müller L, Kilian M, Schuhmann MU, Baumann L, Handrick W (2008). Recurrent posttraumatic meningitis due to nontypable Haemophilus influenzae: case report and review of the literature. Infection.

[6] Martinello RA, Teitelbaum J, Young E, Hostetter MK (2004). Nontypable haemophilus influenzae meningitis in an eleven-year-old. Pediatr Infect Dis J.

[7] Faden H (1991). Meningitis caused by nontypable Haemophilus influenzae in a four-month-old infant. Pediatr Infect Dis J.

[8] Retter ME, Bannatyne RM (1980). Neonatal infection with Haemophilus influenzae biotype III. Can Med Assoc J.

[9] Hershckowitz S, Elisha MB, Fleisher-Sheffer V, Barak M, Kudinsky R, Weintraub Z (2004). A cluster of early neonatal sepsis and pneumonia caused by nontypable Haemophilus influenzae. Pediatr Infect Dis J.

[10] Kay SE, Nack Z, Jenner BM (2001). Meningitis and septicaemia in a child caused by non-typable Haemophilus influenzae biotype III. Med J Aust.

[11] Cuthill SL, Farley MM, Donowitz LG (1999). Nontypable Haemophilus influenzae meningitis. Pediatr Infect Dis J.

[12] Nizet V, Colina KF, Almquist JR, Rubens CE, Smith AL (1996). A virulent nonencapsulated Haemophilus influenzae. J Infect Dis.

[13] Takano C, Seki M, Kim DW (2017). Molecular serotype-specific identification of non-type B Haemophilus influenzae by loop-mediated isothermal amplification. Front Microbiol.

[14] (2019). Reference Range Values for Pediatric Care (2nd Edition).

[15] Peltola H (2000). Worldwide Haemophilus influenzae type b disease at the beginning of the 21st century: global analysis of the disease burden 25 years after the use of the polysaccharide vaccine and a decade after the advent of conjugates. Clin Microbiol Rev.

[16] Ulanova M, Tsang RS (2014). Haemophilus influenzae serotype a as a cause of serious invasive infections. Lancet Infect Dis.

[17] Desai S, Jamieson FB, Patel SN (2015). The epidemiology of invasive Haemophilus influenzae non-serotype B disease in Ontario, Canada from 2004 to 2013. PLoS ONE.

[18] LaClaire LL, Tondella ML, Beall DS, Noble CA, Raghunathan PL, Rosenstein NE, Popovic T (2003). Identification of Haemophilus influenzae serotypes by standard slide agglutination serotyping and PCR-based capsule typing. J Clin Microbiol.

[19] Kim DW, Kilgore PE, Kim EJ, Kim SA, Anh DD, Seki M (2011). Loop-mediated isothermal amplification assay for detection of Haemophilus influenzae type b in cerebrospinal fluid. J Clin Microbiol.

[20] Resman F, Ristovski M, Ahl J (2011). Invasive disease caused by Haemophilus influenzae in Sweden 1997-2009; evidence of increasing incidence and clinical burden of non-type b strains. Clin Microbiol Infect.

[21] Collins S, Litt DJ, Flynn S, Ramsay ME, Slack MP, Ladhani SN (2015). Neonatal invasive Haemophilus influenzae disease in England and Wales: epidemiology, clinical characteristics, and outcome. Clin Infect Dis.

[22] Giufrè M, Cardines R, Accogli M, Cerquetti M (2015). Neonatal invasive Haemophilus influenzae disease and genotypic characterization of the associated strains in Italy. Clin Infect Dis.

[23] Sawardekar KP (2017). Haemophilus influenzae type a meningitis in immunocompetent child, Oman, 2015. Emerg Infect Dis.

[24] Antony S, Kaushik A, Mauriello C, Chatterjee A (2017). Non-type b Haemophilus influenzae invasive infections in North Dakota and South Dakota, 2013-2015. J Pediatric Infect Dis Soc.

[25] Adderson EE, Byington CL, Spencer L (2001). Invasive serotype a Haemophilus influenzae infections with a virulence genotype resembling Haemophilus influenzae type b: emerging pathogen in the vaccine era?. Pediatrics.

[26] Mclntyre PB, Berkey CS, King SM (1997). Dexamethasone as adjunctive therapy in bacterial meningitis: a meta-analysis of randomized clinical trials since 1988. JAMA.

[27] Ogunlesi TA, Odigwe CC, Oladapo OT (2015). Adjuvant corticosteroids for reducing death in neonatal bacterial meningitis. Cochrane Database Syst Rev.

